# Author Correction: ERK and USP5 govern PD-1 homeostasis via deubiquitination to modulate tumor immunotherapy

**DOI:** 10.1038/s41467-025-61049-w

**Published:** 2025-06-20

**Authors:** Xiangling Xiao, Jie Shi, Chuan He, Xia Bu, Yishuang Sun, Minling Gao, Bolin Xiang, Wenjun Xiong, Panpan Dai, Qi Mao, Xixin Xing, Yingmeng Yao, Haisheng Yu, Gaoshan Xu, Siqi Li, Yan Ren, Baoxiang Chen, Congqing Jiang, Geng Meng, Yu-Ru Lee, Wenyi Wei, Gordon J. Freeman, Conghua Xie, Jinfang Zhang

**Affiliations:** 1 Department of Radiation and Medical Oncology, Hubei Key Laboratory of Tumor Biological Behaviors, Hubei Cancer Clinical Study Center, Zhongnan Hospital of Wuhan University; Medical Research Institute, Frontier Science Center of Immunology and Metabolism, Wuhan University, Wuhan, 430071 China; 2 Taikang Center for Life and Medical Sciences, Wuhan University, Wuhan, 430071 China; 3 Department of Medical Oncology, Dana-Farber Cancer Institute, Harvard Medical School, Boston, MA 02115 USA; 4 Department of Biology, University of Copenhagen, Copenhagen, 2100 Denmark; 5 Experiment Center for Science and Technology, Shanghai University of Traditional Chinese Medicine, Shanghai, 201203 China; 6 Department of Colorectal and Anal Surgery, Low Rectal Cancer Diagnosis and Treatment Center, Zhongnan Hospital of Wuhan University, Wuhan, 430071 China; 7 College of Veterinary Medicine, China Agricultural University, Beijing, 100094 China; 8 Institute of Biomedical Sciences, Academia Sinica, Taipei, 115201 Taiwan; 9 Department of Pathology, Beth Israel Deaconess Medical Center, Harvard Medical School, Boston, MA 02115 USA

**Keywords:** Cancer therapy, Tumour immunology, Tumour immunology

Correction to: *Nature Communications* 10.1038/s41467-023-38605-3, published online 19 May 2023

In the version of the article initially published, there were errors in Fig. 6d, Supplementary Figs. 6d and 10b, and the associated Source data.

In Fig. 6d, the image for Gzmb IHC staining following Trametinib treatment was inadvertently duplicated from the corresponding image following Combination treatment. The correct version of this figure has been added to the original article.

In Supplementary Fig. 6d, the western blot showing USP5 in the “IP: PD-1” group was inadvertently duplicated from Supplementary Fig. 6c. The correct version of this figure is now provided online in the updated Supplementary information.

The Source data for Supplementary Fig. 10b was inadvertently duplicated from Supplementary Fig. 10a. We have now provided the corrected dataset, and the corrected Supplementary Fig. 10b is now provided online in the updated Supplementary information.

The corrected Supplementary information and Source data are available alongside the original article. The Supplementary information accompanying this amendment contains the original, incorrect figures for comparison.

## Supplementary information

Original, incorrect figures

