# Supplementary material for: ERK and USP5 govern PD-1 homeostasis via deubiquitination to modulate tumor immunotherapy
Source: Nat Commun. 2023 May 19;14:2859. doi: 10.1038/s41467-023-38605-3 (PMC10199079; doi:10.1038/s41467-023-38605-3)
Supplement: Supplementary file 1 — Supplementary Information [file 41467_2023_38605_MOESM1_ESM.pdf]

Supplementary Information for

ERK and USP5 govern PD-1 homeostasis via deubiquitination to modulate tumor immunotherapy

Xiangling Xiao[1,2,#], Jie Shi[1,2,#], Chuan He[1,2,#], Xia Bu[3], Yishuang Sun[1,2], Minling Gao[1,2], Bolin Xiang[1,2], Wenjun Xiong[1,2], Panpan Dai[1], Qi Mao[1,2], Xixin Xing[1,2], Yingmeng Yao[1,2], Haisheng Yu[1,2], Gaoshan Xu[1,2], Siqi Li[4], Yan Ren[5], Baoxiang Chen[6], Congqing Jiang[6], Geng Meng[7], Yu-Ru Lee[8], Wenyi Wei[9], Gordon J. Freeman[3],
Conghua Xie[1,*], Jinfang Zhang[1,2,*]

[1]Department of Radiation and Medical Oncology, Hubei Key Laboratory of Tumor Biological Behaviors, Hubei Cancer Clinical Study Center, Zhongnan Hospital of Wuhan University; Medical Research Institute, Frontier Science Center of Immunology and Metabolism, Wuhan University, Wuhan 430071, China
[2]Taikang Center for Life and Medical Sciences, Wuhan University, Wuhan 430071, China
[3]Department of Medical Oncology, Dana-Farber Cancer Institute, Harvard Medical School, Boston, MA 02115, USA
[4]Department of Biology, University of Copenhagen, Copenhagen 2100, Denmark
[5]Experiment Center for Science and Technology, Shanghai University of Traditional Chinese Medicine, Shanghai 201203, China
[6]Department of Colorectal and Anal Surgery, Low rectal cancer diagnosis and treatment center, Zhongnan Hospital of Wuhan University, Wuhan, 430071, China
[7]College of Veterinary Medicine, China Agricultural University, Beijing, 100094, China
[8]Institute of Biomedical Sciences, Academia Sinica, Taipei 115201, Taiwan
[9]Department of Pathology, Beth Israel Deaconess Medical Center, Harvard Medical School, Boston, MA 02115, USA
[#]These authors contributed equally
[*]Corresponding author:
chxie_65@whu.edu.cn (C.X.); jinfang_zhang@whu.edu.cn (J.Z.)

This PDF file includes:

Supplementary text
Supplementary Figures 1 to 10

Supplementary Fig. 1

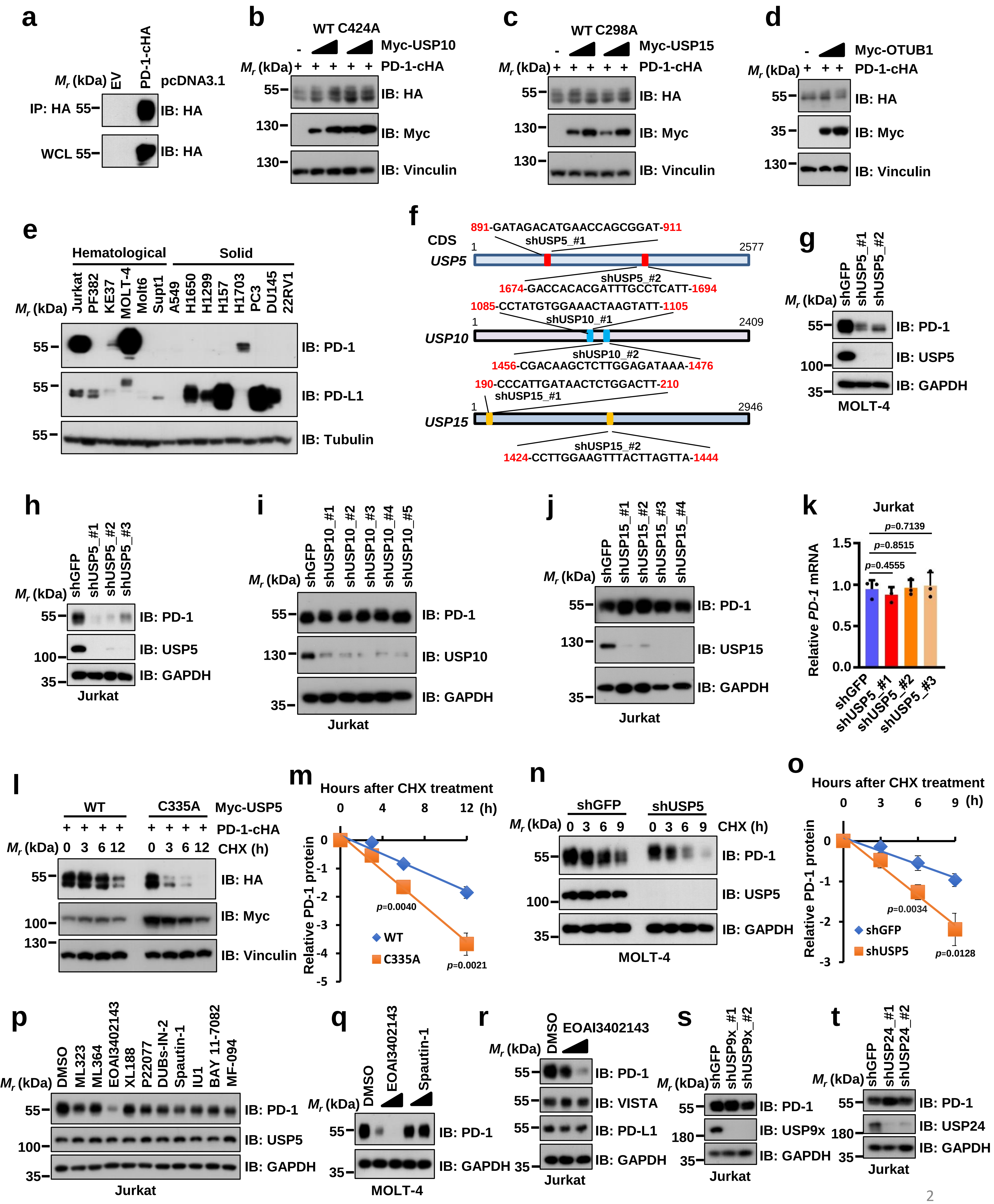

Supplementary Fig. 1 Identifying USP5 as a positive regulator for PD-1.

a Immunoblotting (IB) analysis of whole-cell lysates (WCL) derived from HEK293T cells transfected pcDNA3.1-PD-1-cHA as well as the empty vector (EV) as control. After 36 hours (h) post-transfection, cells were harvested for immunoprecipitation (IP) using anti-HA-agarose, respectively..

b-d IB analysis of WCL derived from HEK293T cells co-transfected pcDNA3.1-PD-1-cHA with indicated deubiquitinase constructs, USP10 (**b**), USP15 (**c**) and OTUB1 (**d**).

e IB analysis of WCL derived from different cancer cell lines including hematological and solid cancer cell lines.

f The graphic illustrations to show the shRNA-targeting sequence and its location on each of *USP5*, *USP10*, or *USP15* genes, respectively

g IB analysis of WCL derived from MOLT-4 cells stably expressing shUSP5 and shGFP. Cells were selected with puromycin (1.5 μg/mL) for three days before harvesting.

h-j IB analysis of WCL derived from Jurkat cells infected with lentiviruses expressing shUSP5 (**h**), shUSP10 (**i**), or shUSP15 (**j**) as well as shGFP as a negative control. Cells were stimulated with phytohemagglutinin A (PHA, 150 ng/mL) and selected with puromycin (2 μg/mL) for three days before harvesting.

k The mRNA level of *PD-1* was measured using quantitative reverse transcription PCR (qRT-PCR) for (**h**). Data were presented as mean ± S.D. n = 3 biologically independent samples per group. Two-tailed unpaired t-test.

l-o IB analysis of WCL derived from HEK293T cells transfected with indicated constructs (**l**) or MOLT-4 cells stably expressing shUSP5 as well as shGFP (**n**). Cells were treated with 200 μg/mL cycloheximide (CHX) for indicated time points before harvesting. PD-1 band intensity was quantified by Image J, which was normalized to Vinculin/GAPDH and then compared to the t = 0 timepoint (**m, o**). Data were presented as mean ± S.D. n = 3 biologically independent samples per group. Two-tailed unpaired t-test.

p IB analysis of WCL derived from Jurkat cells, which were stimulated with PHA (150 ng/mL) for three days and treated with indicated various deubiquitinase inhibitors (1.5 μM) for 8 h before harvesting.

q IB analysis of WCL derived from MOLT-4 cells treated with different concentrations of the USP5 inhibitor (1 μM or 2.5 μM EOAI3402143) or USP10 inhibitor (1 μM or 2.5 μM Spautin-1) for 8 h before harvesting.

r IB analysis of WCL derived from Jukat cells stimulated with PHA (150 ng/mL) for three days and treated with different concentrations of EOAI3402143 (1 or 1.5 μM) for 8 h before harvesting.

s, t IB analysis of WCL derived from Jurkat cells infected with indicated lentiviruses expressing shRNAs against USP9x (**s**) or USP24 (**t**). Cells were stimulated with PHA (150 ng/mL) for three days and selected with puromycin (2 μg/mL) for 3 days before harvesting.

All data are representative of independent experiments except for **h**, **l**, and **n**. Source data are provided as a Source Data file.

Supplementary Fig. 2

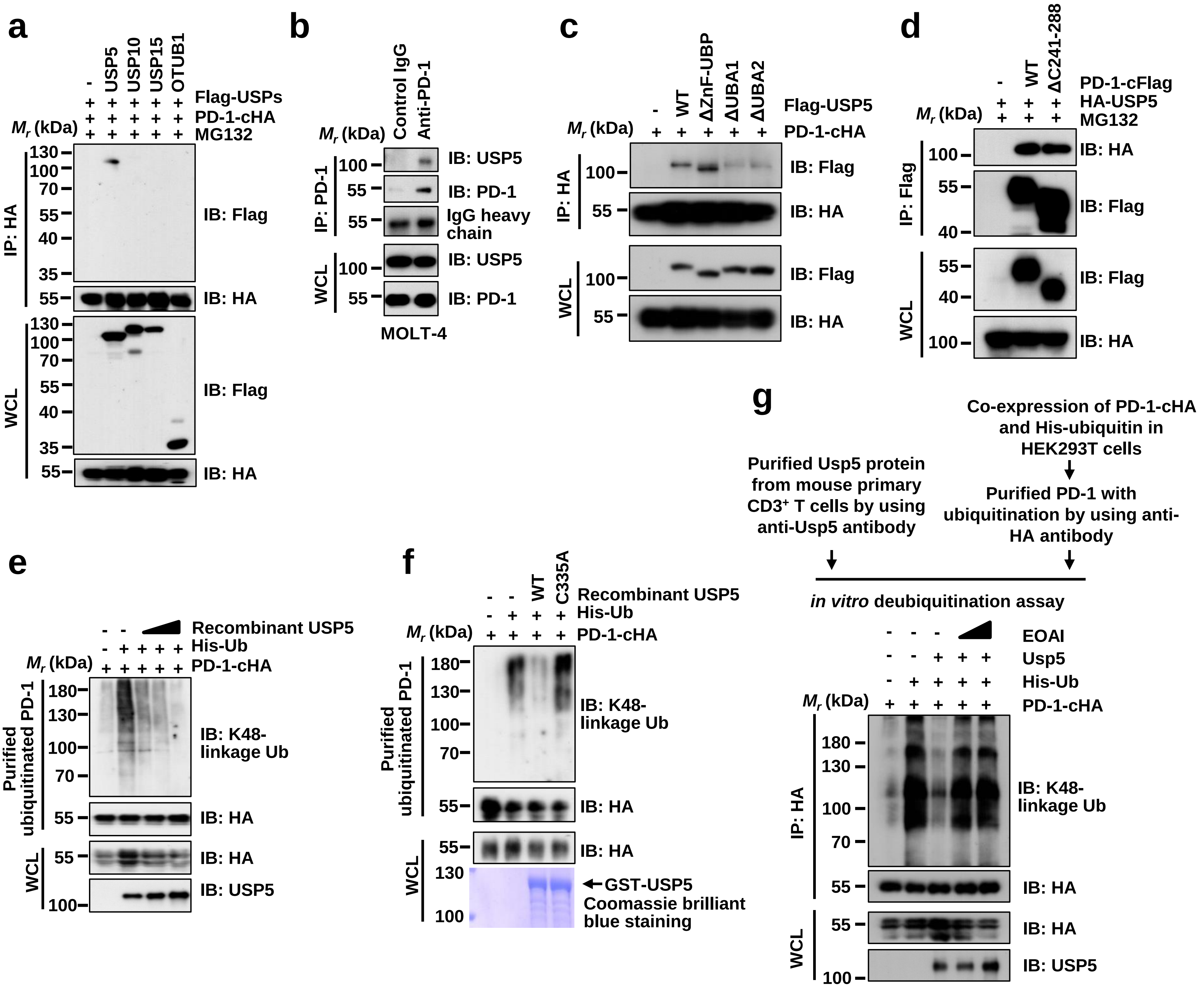

Supplementary Fig. 2 USP5 interacts with PD-1 and deubiquitinates PD-1.

a IB analysis of WCL and anti-HA IPs from HEK293T cells transfected with the indicated constructs. Cells were treated with 10 μM MG132 for 12 h before harvesting.

b IB analysis of WCL and anti-PD-1 IPs derived from MOLT-4 cells.

c IB analysis of WCL and anti-HA IPs derived from HEK293T cells co-transfected PD-1-cHA with Flag-USP5 WT or different deletion mutants.

d IB analysis of WCL and anti-Flag IPs derived from HEK293T cells transfected with indicated constructs.

e, f *In vitro* deubiquitination assay, HEK293T cells transfected with His-ubiquitin and PD-1-cHA were treated with 10 μM MG132 for 12 h. Ubiquitinated PD-1 was purified with anti-HA beads and was incubated without or with purified recombinant His-USP5 (**e**) or GST-USP5 WT/C335A (**f**). IB analysis with indicated antibodies.

g *In vitro* deubiquitination assays of incubating the purified Usp5 proteins from mouse primary $CD3^+$ T cell with ubiquitinated PD-1 from HEK293T cells that transfected with PD-1-cHA and His-ubiquitin. The mixtures were incubated at 30° C for 6 h with/without different concentrations of EOAI3402143 and analyzed by IB. All data are representative of two independent experiments. Source data are provided as a Source Data file.

Supplementary Fig. 3

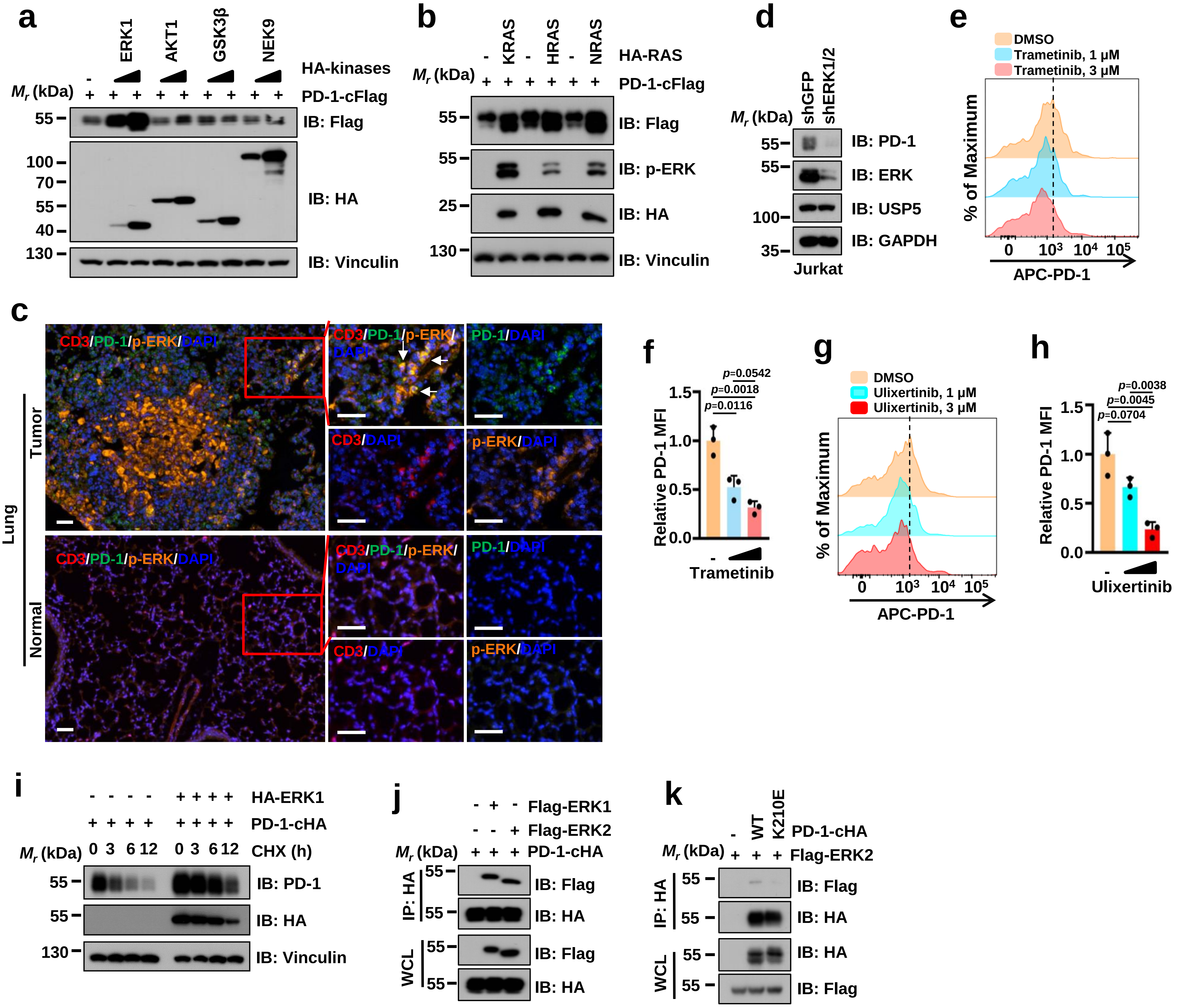

Supplementary Fig. 3 ERK phosphorylates PD-1 at Thr234 to stabilize PD-1.

a, b IB analysis of WCL derived from HEK293T cells transfected with indicated constructs.

c Representative multiplex immunohistochemistry (mIHC) images of CD3 (red), PD-1 (green), phosphor-ERK at Thr202/Try204 (p-ERK, orange), and DAPI nuclear staining (blue) in mouse lung tumor (upper panels) or normal (lower panels) sections. n = 4 mice each group. Scale bars, 50 μm.

d IB analysis of WCL derived from Jurkat cells stably expressing shERK1/2 or shGFP.

e, f Cell surface PD-1 on Jurkat cells treated with Trametinib (1 μM or 3 μM) for 24 h was analyzed by flow cytometry (**e**). The relative mean fluorescence intensity (MFI) of PD-1 on the surface of Jurkat cells was quantified (**f**). Data were presented as mean ± S.D. n = 3 biologically independent samples per group. Two-tailed unpaired *t*-test.

g, h Cell surface PD-1 on Jurkat cells treated with Ulixertinib (1 μM or 3 μM) for 24 h was analyzed by flow cytometry (**g**). The relative mean fluorescence intensity (MFI) of PD-1 on surface of Jurkat cells was quantified (**h**). Data were presented as mean ± S.D. n = 3 biologically independent samples per group. Two-tailed unpaired *t*-test.

i IB analysis of WCL derived from HEK293T cells transfected with PD-1 and ERK1 as indicated. The cell was treated with 200 μg/mL cycloheximide (CHX) at indicated time points before harvesting.

j IB analysis of WCL and anti-HA IPs derived from HEK293T cells co-transfected PD-1-cHA with Flag-ERK1 or ERK2 as indicated.

k IB analysis of WCL and anti-HA IPs derived from HEK293T cells co-transfected Flag-ERK2 with PD-1 WT or K210E mutant as indicated.

For **a**, **b**, **d**, **i**, **j**, and **k**, data are representative of at two independent experiments. Source data are provided as a Source Data file.

Supplementary Fig. 4

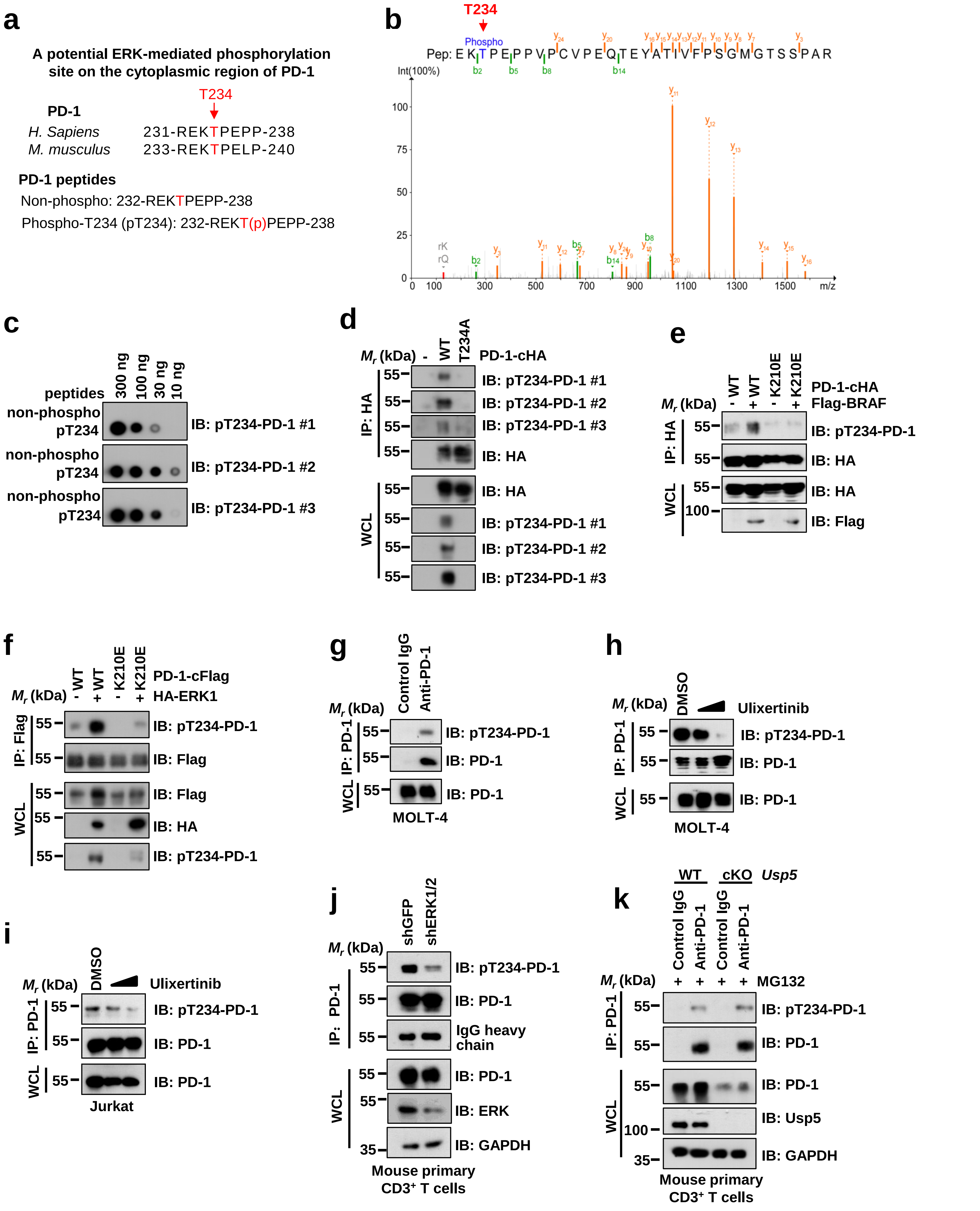

Supplementary Fig. 4 ERK phosphorylates PD-1 at Thr234.

a Protein sequence alignment for illustrating synthesized PD-1 peptides that generate anti-phospho-T234-PD-1 antibody.

b Mass spectrometry to identify the phosphorylation site on PD-1. PD-1 protein was purified with anti-HA beads derived from HEK293T cells co-transfected with PD-1-cHA and Flag-ERK1. Mass spectrum for peptide-containing PD-1 Thr234 phosphorylation was shown. The b-type product ions (b3) are marked on the spectrum and illustrated along the peptide sequence shown on top of the spectrum.

c Dot blot analysis to show that generated pT234-PD-1 antibodies specifically recognize the phosphorylated T234 PD-1 peptides *in vitro*.

d IB analysis of WCL and anti-HA IPs derived from HEK293T cells transfected with indicated constructs to demonstrate that generated PD-1-pT234 antibodies specifically recognize PD-1 WT, but not T234A mutant in cells.

e, f IB analysis of WCL and anti-HA or Flag IPs derived from HEK293T cells transfected with indicated constructs.

g IB analysis of WCL and anti-PD-1 IPs derived from MOLT-4 cells with indicated antibodies.

h, i IB analysis of WCL and anti-PD-1 IPs derived from MOLT-4 (**h**) or Jurkat (**i**) cells. Cells were treated with 0.5 or 1 μM Ulixertinib for 24 h.

j IB analysis of WCL and anti-PD-1 IPs derived from mouse primary $CD3^+$ T cells stably expressing shGFP or shUsp5. Cells were stimulated with anti-CD3/CD28 (2 μg/mL) for 48 h.

k IB analysis of WCL and anti-PD-1 IPs derived from mouse primary *Usp5*$^{fl/fl}$ (WT) or *Usp5*$^{fl/fl}$ *Cd4*-Cre (cKO) $CD3^+$ T cells. Cells were stimulated with anti-CD3/CD28 (2 μg/mL) for 48 h and treated with MG132 (5 μM) for 4 h.

All data are representative of two independent experiments. Source data are provided as a Source Data file.

Supplementary Fig. 5

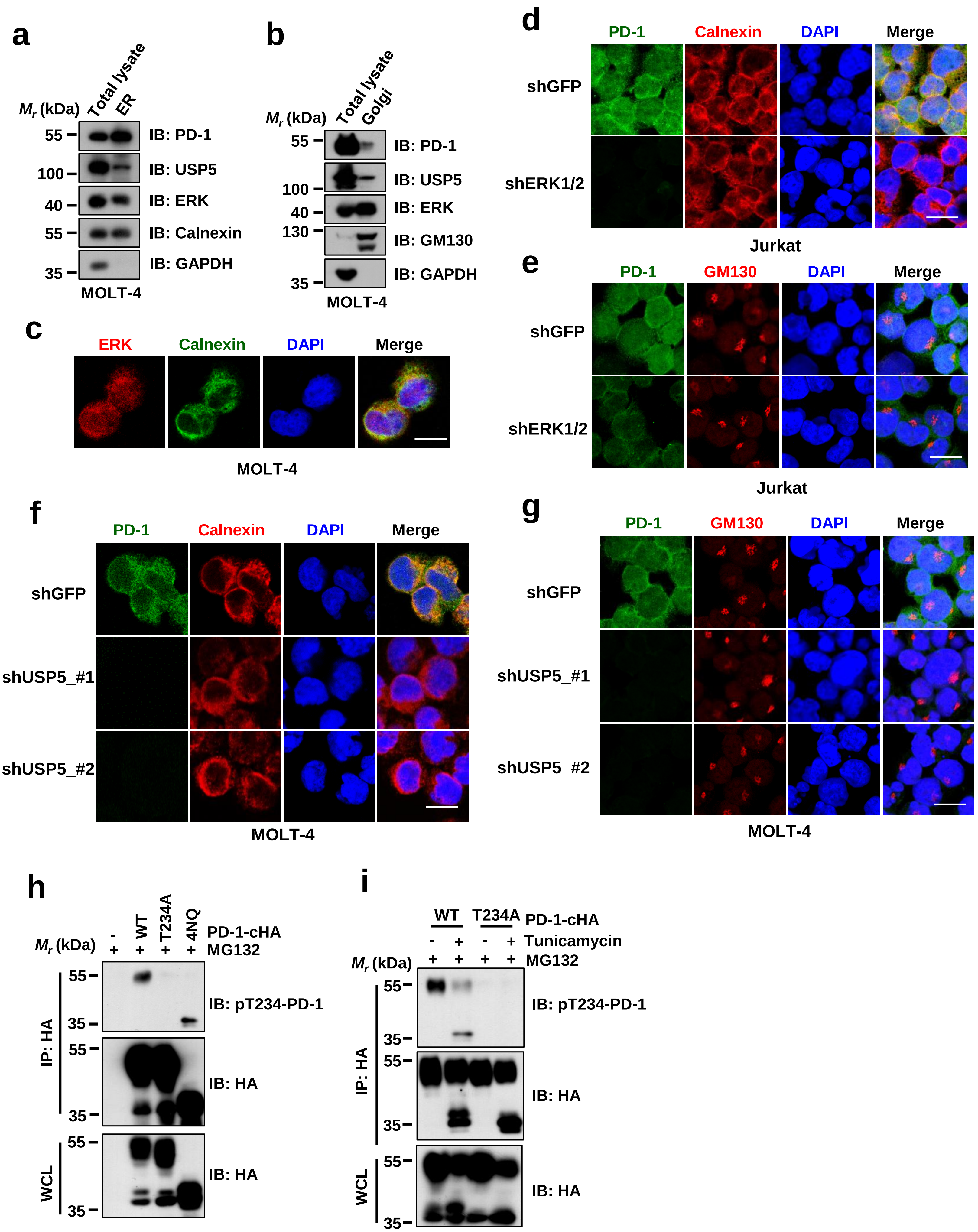

Supplementary Fig. 5 Knockdown of *ERK* or *USP5* disrupts PD-1 localization in the endoplasmic reticulum (ER) and Golgi apparatus.

a, b IB analysis of purified ER (**a**) or Golgi apparatus (**b**) from MOLT-4 cells.

c Immunofluorescence (IF) staining of calnexin (green), ERK (red), and DAPI (blue) in MOLT-4 cells. Scale bars, 20 μm.

d, f IF staining of PD-1 (green), calnexin (red), and DAPI (blue) in Jurkat or MOLT-4 cells stably expressing shGFP, shERK1/2 (**d**), or shUSP5 (**f**). Scale bars, 20 μm.

e, g IF staining of PD-1 (green), GM130 (red), and DAPI (blue) in Jurkat or MOLT-4 cells stably expressing shGFP, shERK1/2 (**e**) or shUSP5 (**g**). Scale bars, 20 μm.

h IB analysis of WCL and anti-HA IPs derived from HEK293T cell lysates with ectopic expression of PD-1-cHA WT, T234A, or 4NQ (N49Q/N58Q/N74Q/N116Q). Cells were treated with 10 μM MG132 for 12h before harvesting.

i IB analysis of WCL and anti-HA IPs derived from HEK293T cell lysates with ectopic expression of PD-1-cHA WT or T234A. Cells were treated with 5 μg/mL Tunicamycin and 10 μM MG132 for 12 h before harvesting.

All data are representative of two independent experiments. Source data are provided as a Source Data file.

Supplementary Fig. 6

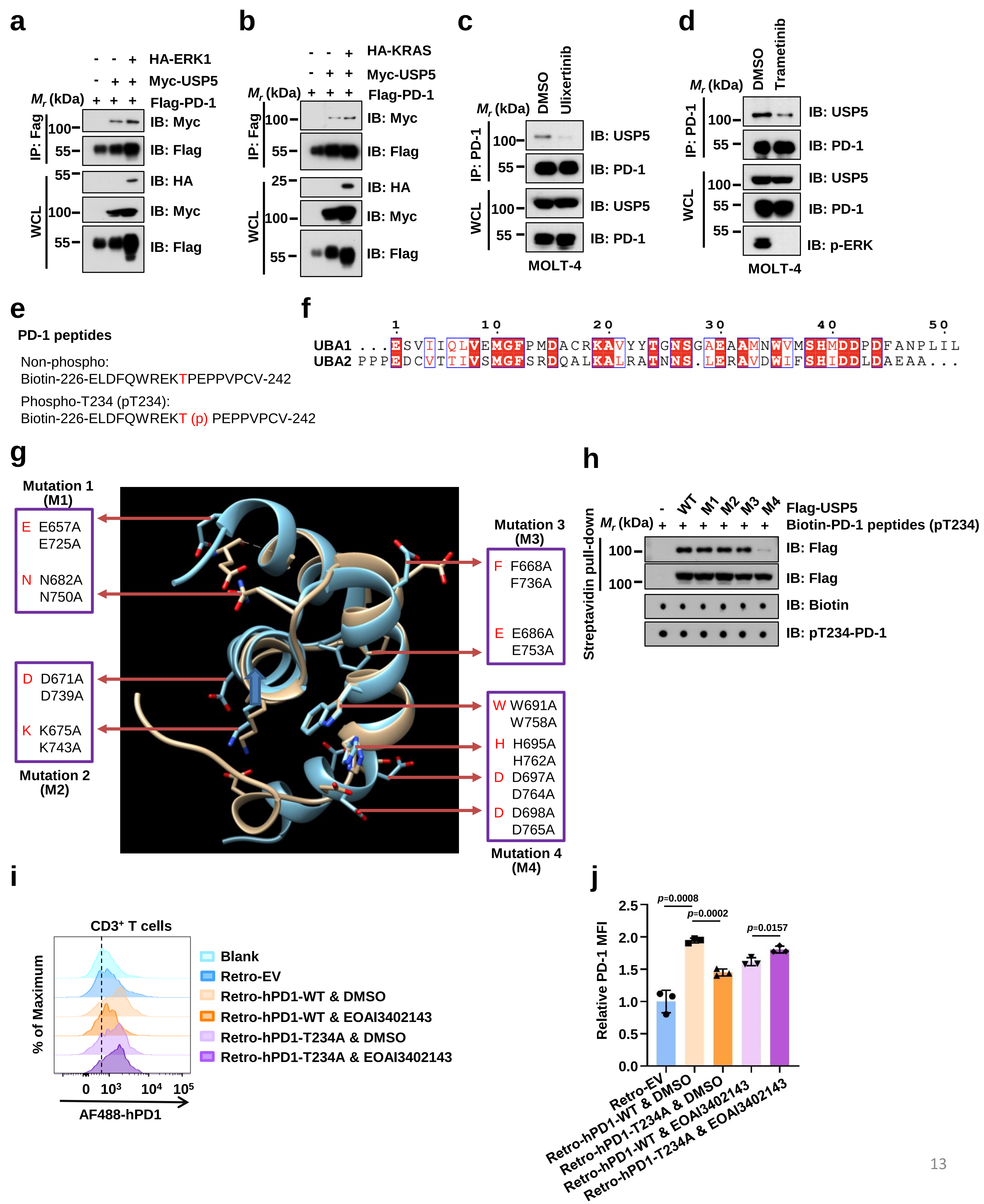

Supplementary Fig. 6 ERK-mediated Thr234 phosphorylation of PD-1 promotes its interaction with USP5.

a, b IB analysis of WCL and anti-Flag IPs from HEK293T cells transfected with indicated constructs.

c, d IB analysis of WCL and anti-PD-1 IPs derived from MOLT-4 cells. Cells were treated with Ulixertinib (0.5 μM) for 24 h or Trametinib (0.5 μM) for 24 h as indicated and MG132 (5 μM) for 6 h before harvesting.

e Protein sequence alignment for illustrating synthesized biotin-labeled synthetic PD-1 peptides.

f The sequence alignment of USP5 UBA1 and UBA2 identified the conserved amino acid residues between the two domains.

g The diagram of aligned USP5 UBA1 (light blue) and UBA2 (light yellow) crystal structure (PDB code: 5UM6) was illustrated using the Chimera software. Conserved amino acid residues on the surface of UBA1 and UBA2 structures were highlighted with arrows and were divided into four groups based on their positions in the structure.

h 3 μg of biotin-labeled synthetic pT234-PD-1 peptides were incubated with WCL of HEK293T cells transfected with Flag-USP5 WT and different mutants as indicated. Streptavidin beads were added to perform pull-down assays and precipitations were analyzed with IB. Dot blot were used to identify biotin-labeled synthetic PD-1 peptides.

i, j Mouse primary T cells were re-introduced with human PD-1 WT or T234A using the retro-virus expression system. The cells were treated with DMSO or EOAI3402143 (2 μM) for 6 h before harvesting for FACS analysis using the human PD-1 antibody that specifically recognizes human, but not mouse PD-1. Data were presented as mean ± S.D. n = 3 biologically independent samples per group. Two-tailed unpaired *t*-test.

For **a**, **b**, **c**, **d** and **h**, data are representative of two independent experiments. Source data are provided as a Source Data file.

Supplementary Fig. 7

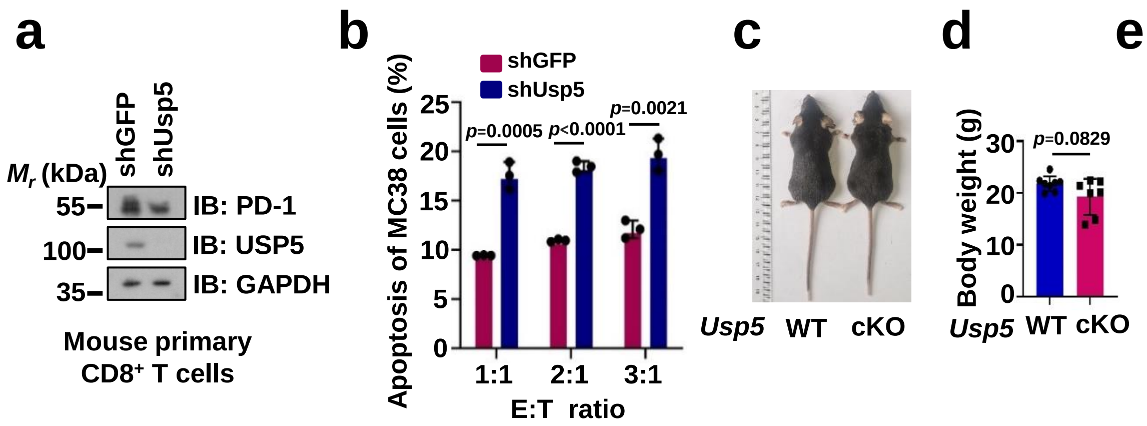

e

Usp5 genotypes	*Usp5*flox/flox	*Usp5*flox/flox; *Cd4l+*	*Usp5*flox/+; *Cd4l+*	*Usp5*flox/+
% of expected embryos	25%	25%	25%	25%
% of observed pups at P0	23.9% n = 59	25.9% n = 64	26.7% n = 66	23.5% n = 58

f

sex ratio	♀	♂
% of expected embryos	50%	50%
% of observed pups at P0	51.8% n = 128	48.2% n = 119

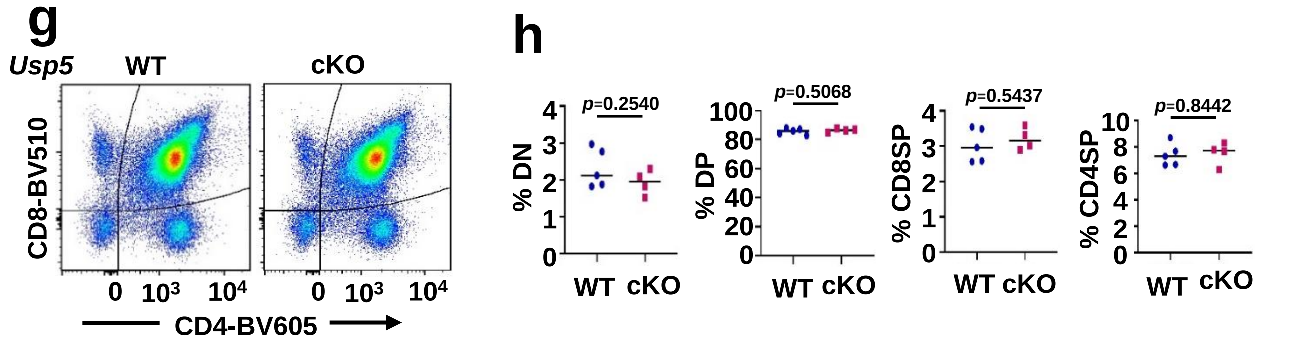

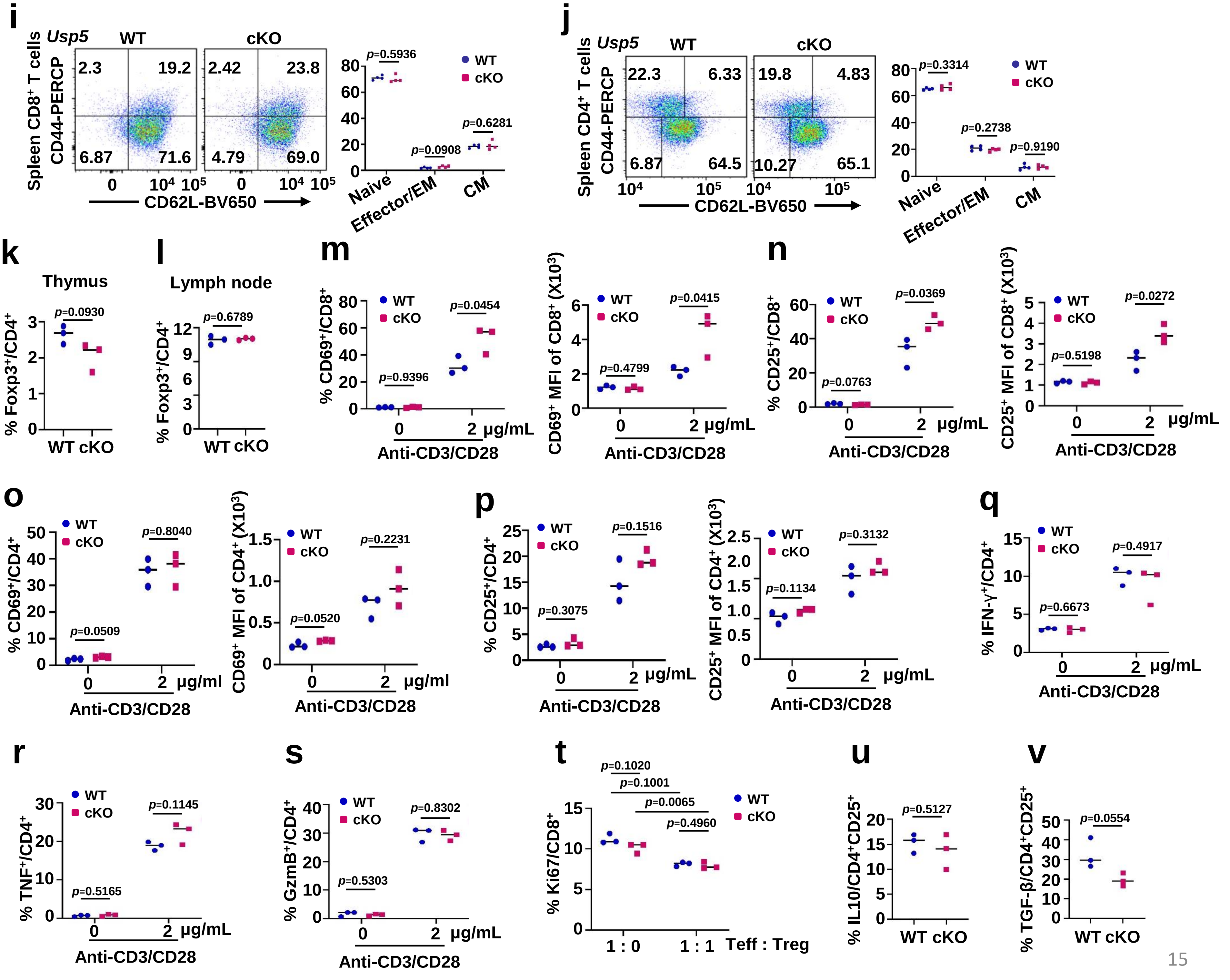

Supplementary Fig. 7 Phenotypes and T cell development of *Usp5* conditional knockout (*Usp5* cKO) mice.

a IB analysis of WCL derived from the OT-1 $CD8^+$ T cells stably expressing shUsp5 or shGFP

b *In vitro* $CD8^+$ T cell-mediated tumor cell-killing assays. MC38 cell viability was analyzed after co-culture with different activated OT-1 $CD8^+$ T effector cells (E) treated with shGFP/shUSP5 and OVA-pulsed MC38 tumor target cells (T) (E:T ratio). The bar plots represent the relative apoptosis cell number of MC38 target cells n = 3 biologically independent samples per group.

c, d General appearance (**c**) and body weights (**d**) among *Usp5* cKO and WT mice aged 9 weeks. WT: n = 8 mice, cKO: n = 7 mice.

e, f Mendelian inheritance ratios from the interbreeding of $Usp5^{flox/+}Cd4^{/+}$ and $Usp5^{flox/flox}$ mice, the number and birth ratio of four genotypes of mice were shown (**e**). The number and sex ratio of mice were shown (**f**).

g, h The percentages of $CD4^-CD8^-$ double-negative (DN), $CD4^+CD8^+$ double-positive (DP), $CD8^+$ single-positive (CD8SP) and $CD4^+$ single-positive (CD4SP) cells out of the total number of thymocytes from WT or *Usp5* cKO mice. Representative flow cytometry plots (**g**) and quantification (**h**). (8 weeks old, WT: n = 5 mice, cKO: n = 4).

i, j The percentages of naive ($CD44^{low}CD62L^{high}$), effector/effector memory ($CD44^{high}CD62L^{low}$, effector/EM), and central memory ($CD44^{high}CD62L^{high}$, CM) cells of total peripheral T cells isolated from spleens of WT and *Usp5* cKO mice. (8 weeks old, WT: n = 4 mice, cKO: n = 4).

k, l Quantification of $Foxp3^+$ T cells represented as the percentage of $CD4^+$ T cells in the thymus (**k**) or lymph node (**l**) derived from WT or *Usp5* cKO mice. n = 3 mice per group.

m, n Quantification of $CD69^+$ (**m**) or $CD25^+$ (**n**) T cells represented as the percentage of $CD8^+$ T cells in spleen derived from WT or *Usp5* cKO mice. Cells were stimulated with anti-CD3/CD28 (5 h for $CD69^+$ T cell analysis; 13 h for $CD25^+$ T cell analysis). n = 3 mice per group.

o, p Quantification of $CD69^+$ (**o**) or $CD25^+$ (**p**) T cells represented as the percentage of $CD4^+$ T cells in spleen derived from WT or *Usp5* cKO mice. Cells were stimulated with anti-CD3/CD28 (5 h for $CD69^+$ T cell analysis; 13 h for $CD25^+$ T cell analysis). n = 3 mice per group.

q-s The production of IFNγ (**q**), TNF (**r**), or GzmB (**s**) in WT and *Usp5* cKO $CD8^+$ T cells, which was stimulated with anti-CD3/CD28 (0 or 2 μg/mL) for 96 h and treated with Brefeldin A (BFA, 5 μg/mL) for 5 h. n = 3 biologically independent samples.

t-v $CD8^+$ T cells were co-cultured with $CD4^+CD25^+$ T cells isolated from WT or *Usp5* cKO for 4 days in the presence of plate-bound 1 μg/mL anti-CD3/CD28. The suppressive activity of $CD4^+CD25^+$ T cells was determined by measuring the proliferation of activated $CD8^+$ T cells staining with APC-Ki67 (**t**). At the same time, the inhibitory cytokine production of IL10 (**u**) and TFG-β (**v**) in WT and *Usp5* cKO $CD4^+CD25^+$ T cells was also measured. n = 3 biologically independent samples.

For **b**, **d**, **h-v**, Data were presented as mean ± S.D. Two-tailed unpaired *t*-test. For **a**, data are representative of three independent experiments. Source data are provided as a Source Data file.

Supplementary Fig. 8

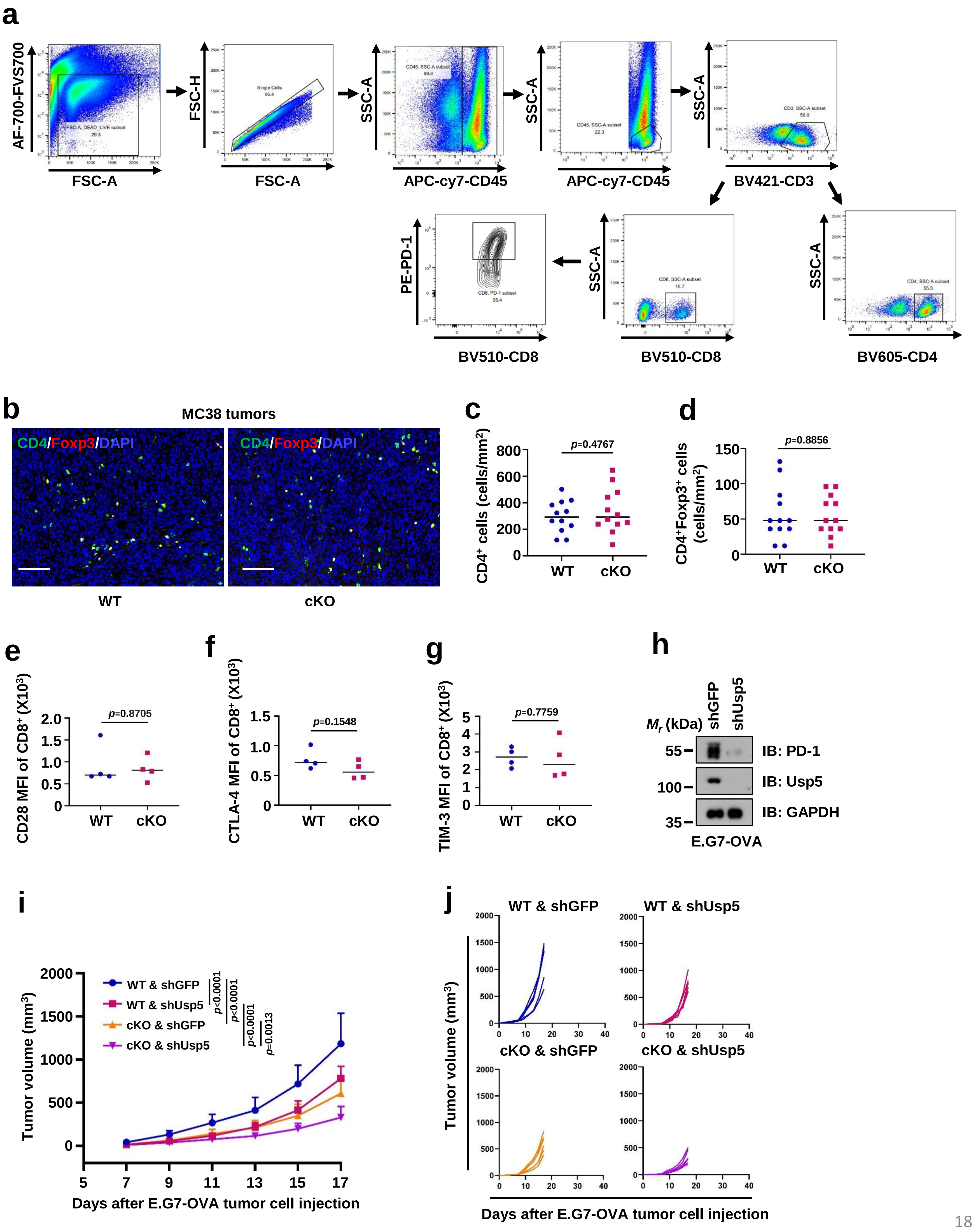

Supplementary Fig. 8 The infiltrating $CD4^+$ T cells and Treg cells are not significantly different in tumors derived from WT and *Usp5* cKO mice.

a The representative FACS gating strategies for analyzing tumor-infiltrating immune cells and PD-1^+CD8^+ T cells. Cells were first gated by FSC-A/AF700-FVS700 to eliminate dead cells and gated by FSC-A and FSC-H to eliminate non-singlet cells**.** $CD8^+$ cells were gated for PD-1 expression analysis. This gating strategy is applicable to analyzing all tumor-infiltrating leukocytes.

b-d Representative mIHC images of CD4 (green), Foxp3 (red), and DAPI nuclear staining (blue) in MC38 tumors derived from WT or *Usp5* cKO mice (**b**). Quantification for $CD4^+$ (**c**) and $CD4^+Foxp3^+$ (**d**) cells were counted from three randomly selected high-power fields from each section. Scale bar: 100 μm. n = 4 mice per group.

e-g Flow cytometric analysis of CD28 (**e**), CTLA-4 (**f**), and TIM-3 (**g**) in infiltrating $CD8^+$ T cells of tumors isolated from WT or *Usp5* cKO mice. n = 4 mice per group.

h IB analysis of WCL derived from E.G7-OVA cells infected with lentiviruses expressing shUSP5 as well as shGFP as a negative control.

i, j Tumor growth of E.G7-OVA (shGFP or shUsp5) cells was assessed in WT or *Usp5* cKO mice (**i**). Tumor volumes of mice were measured every two days and plotted individually (**j**). n = 6 mice per group. Data were presented as mean ± S.D. Two-way ANOVA.

For **c-g**, Data were presented as mean ± S.D. Two-tailed unpaired *t*-test. For **h**, data are representative of three independent experiments. Source data are provided as a Source Data file.

Supplementary Fig. 9

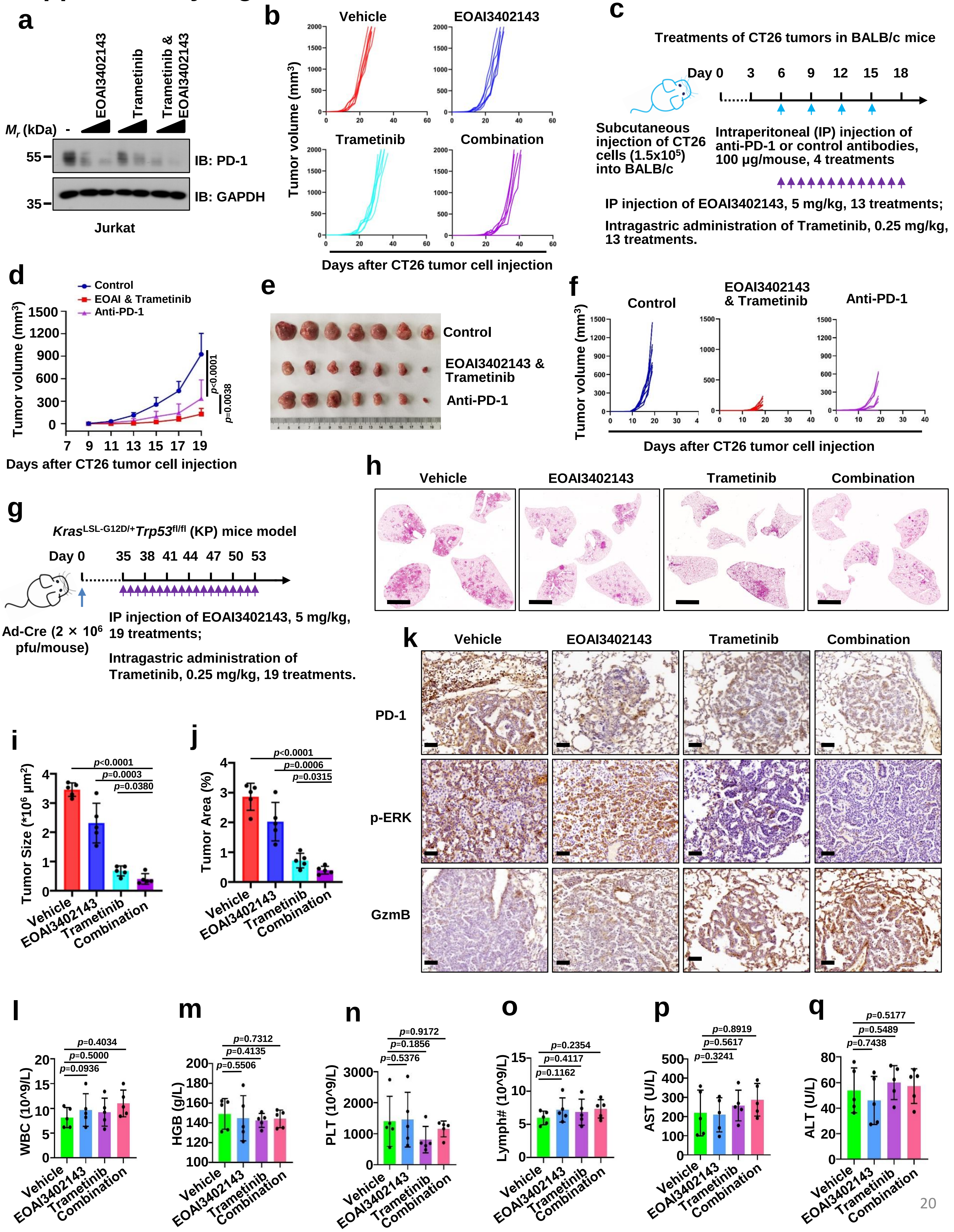

Supplementary Fig. 9 Co-targeting USP5 and ERK signaling has an additive effect on reducing PD-1 and suppressing tumor growth.

a IB analysis of WCL derived from lysates of Jurkat cells treated with indicated EOAI3402143 (1 or 2 μM) for 8 h or Trametinib (1 or 3 μM) for 24 h.

b BALB/c mice bearing CT26 implanted tumors were enrolled in different treatment groups as indicated. Tumor volumes of mice treated with control vehicle, EOAI3402143, Trametinib, or combined were measured every 2 days and plotted individually. n = 6 mice per group.

c A schematic treatment plan for BALB/c mice bearing subcutaneous CT26 tumors. Mice were subcutaneously implanted with 1.5×10^5 CT26 cells and treated with control vehicle, USP5 inhibitor EOAI3402143 combination with Trametinib, or anti-PD-1, respectively.

d-f Tumor growth of CT26 cells in immunocompetent BALB/c mice with indicated treatments (**d**). Images of endpoint subcutaneous tumors (**e**). Tumor volumes of mice treated with control antibody/vehicle, the combination of EOAI3402143 & Trametinib, or anti-PD-1 were measured every two days and plotted individually (**f**). n = 7 mice per group. Data were presented as mean $\pm$ S.D. Two-way ANOVA.

g A scheme of combinational therapy of USP5 inhibitor and MEK inhibitor in $\textit{Kras}^{\text{LSL-G12D/+}}\textit{Trp53}^{\text{fl/fl}}$ (KP) mice bearing induced autochthonous lung cancers. KP mice were intranasally injected with Cre-expressing adenovirus (Ad-Cre, 2×10^6 pfu/mouse) for 35 days, followed by intraperitoneal injection or intragastrical administration of vehicle, EOAI3402143, Trametinib, or combination.

h-j Representative images of HE staining in tumor-burdened lungs of KP mice. Scale bars represent 3 mm (**h**). Tumor sizes (**i**) and tumor areas (**j**) were analyzed with indicated treatments. n = 5 mice per group.

k Representative images from immunohistochemical (IHC) staining analysis of PD-1, p-ERK and Granzyme B (GzmB) in lung tumor sections of KP mice treated as indicated. Scale bars: 50 μm.

l-q There was no significant difference in aspects of blood general examination, aspartate amino transferase (AST) and alanine amino transferase (ALT) among different treatment groups. n = 5 mice per group.

For **i**, **j**, **l-q**, Data were presented as mean ± S.D. Two-tailed unpaired *t*-test. For **a** and **k,** data are representative of three independent experiments. Source data are provided as a Source Data file.

Supplementary Fig. 10

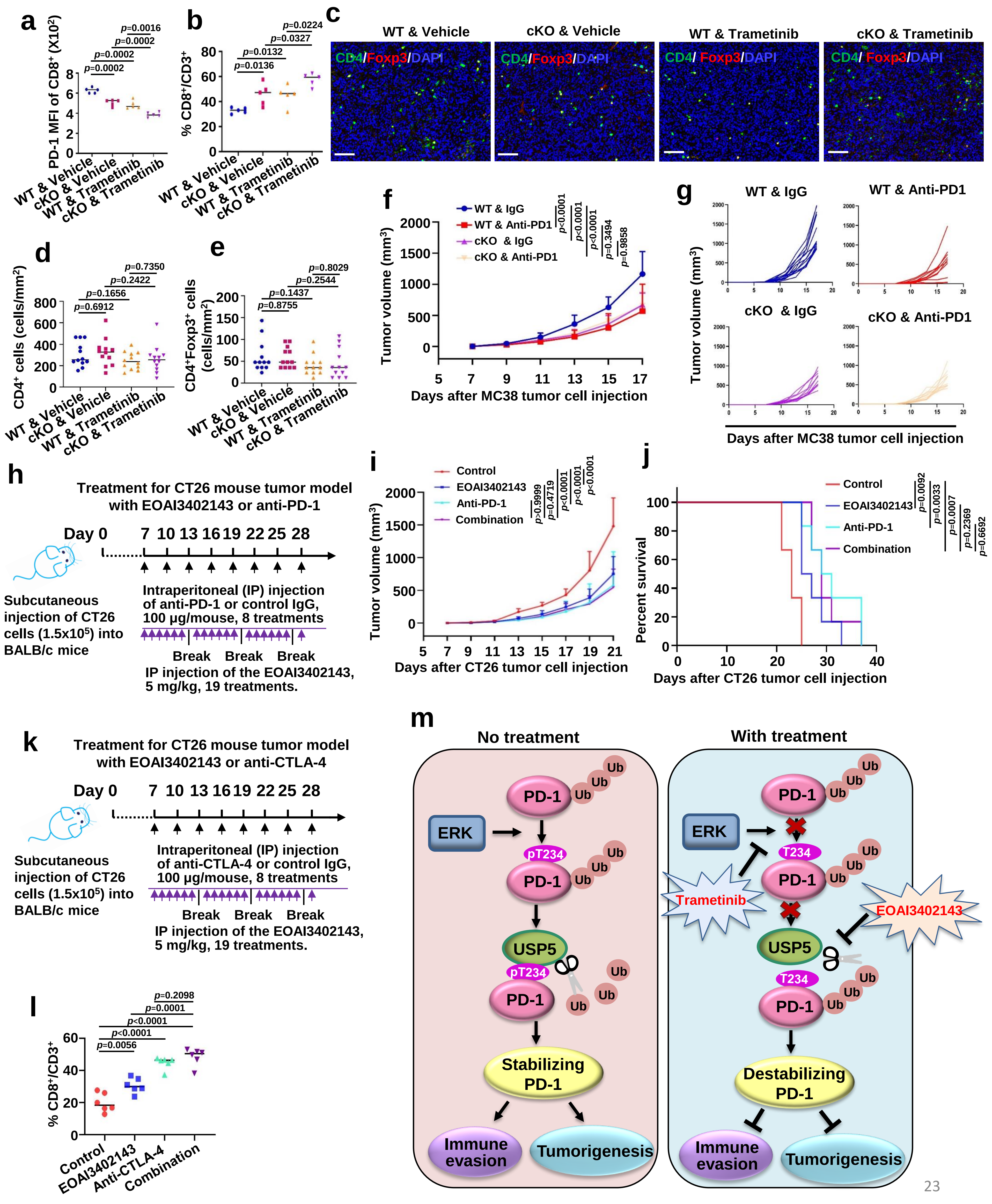

Supplementary Fig. 10 USP5 inhibition in combination with anti-PD-1 does not have an additive effect on suppressing tumor growth.

a, b Quantification of flow cytometry results of PD-1 MFI in tumor-infiltrating $CD8^+$ T cells and $CD8^+$ T cells represented as the percentage of tumor-infiltrating $CD3^+$ T cells in CT26 tumors after indicated treatments. n = 5 each group.

c-e Representative mIHC images of CD4 (green), Foxp3 (red), and DAPI nuclear staining (blue) in MC38 tumors derived from WT or *Usp5* cKO mice (**c**). Quantification for $CD4^+$ (**d**) and $CD4^+Foxp3^+$ (**e**) cells were counted from three randomly selected high-power fields from each section. Scale bar: 100 μm. n = 4 mice per group.

f, g The growth of MC38 tumors in WT or *Usp5* cKO mice with indicated treatments (**f**). Tumor volumes of mice treated with control antibody/vehicle or anti-PD-1 were measured every two days and plotted individually (**g**). n = 10 mice per group. Data were presented as mean ± S.D. Two-way ANOVA.

h A schematic treatment plan for BALB/c mice bearing subcutaneous CT26 tumors. Mice were subcutaneously implanted with 1.5×10^5 CT26 cells and treated with control vehicle, EOAI3402143, anti-PD-1, or combined treatment, respectively.

i, j The growth of CT26 tumors in immunocompetent BALB/c mice with indicated treatments (**i**). Kaplan-Meier survival curves for BALB/c mice bearing CT26 tumors with indicated treatments (**j**). n = 6 mice per group. Data were presented as mean ± S.D. Two-way ANOVA for (**i**). Log-rank test for (**j**).

k A schematic treatment plan for BALB/c mice bearing subcutaneous CT26 tumors. Mice were subcutaneously implanted with 1.5×10^5 CT26 cells and treated with control vehicle, EOAI3402143, anti-CTAL-4, or combined treatment, respectively.

l Quantification of flow cytometry results of $CD8^+$ T cells represented as the percentage of tumor-infiltrating $CD3^+$ T cells in CT26 tumors after indicated treatments. n = 6 each group.

m An illustration to describe the mechanism that USP5/ERK regulates PD-1 stability in a phosphorylation and deubiquitination-dependent manner as well as highlight the combined therapeutic strategies to inhibit immune evasion and tumorigenesis. The ERK-mediated phosphorylation on PD-1 at T234 site promotes USP5-mediated deubiquitination of PD-1 and stabilizes PD-1 in $CD8^+$ T cells, resulting in immune evasion and tumorigenesis (left panel). However, inhibition of USP5 or ERK can reverse this process to destabilize PD-1 and activate $CD8^+$ T cells, which enhances the efficacy of anti-CTLA-4 immunotherapy (right panel).

For **a**, **b**, **d**, **e**, **l**, Data were presented as mean ± S.D. Two-tailed unpaired *t*-test. Source data are provided as a Source Data file.
