# Supplementary material for: ERK and USP5 govern PD-1 homeostasis via deubiquitination to modulate tumor immunotherapy
Source: Nat Commun. 2023 May 19;14:2859. doi: 10.1038/s41467-023-38605-3 (PMC10199079; doi:10.1038/s41467-023-38605-3)
Supplement: Supplementary file 3 — Description of Additional Supplementary Files [file 41467_2023_38605_MOESM3_ESM.pdf]

Description of Additional Supplementary Files

Supplementary Data 1: MS results of anti-HA immuno-complex from WCL with PD-1 or EV sample.
