# Supplementary material for: ERK and USP5 govern PD-1 homeostasis via deubiquitination to modulate tumor immunotherapy
Source: Nat Commun. 2023 May 19;14:2859. doi: 10.1038/s41467-023-38605-3 (PMC10199079; doi:10.1038/s41467-023-38605-3)
Supplement: Supplementary file 5 — Reporting Summary [file 41467_2023_38605_MOESM5_ESM.pdf]

nature portfolio

Corresponding author(s): Jinfang Zhang

Last updated by author(s): May 4, 2023

Reporting Summary

Nature Portfolio wishes to improve the reproducibility of the work that we publish. This form provides structure for consistency and transparency in reporting. For further information on Nature Portfolio policies, see our Editorial Policies and the Editorial Policy Checklist.

Statistics

For all statistical analyses, confirm that the following items are present in the figure legend, table legend, main text, or Methods section.

n/a	Confirmed	
☐	☒	The exact sample size (*n*) for each experimental group/condition, given as a discrete number and unit of measurement
☐	☒	A statement on whether measurements were taken from distinct samples or whether the same sample was measured repeatedly
☐	☒	The statistical test(s) used AND whether they are one- or two-sided<br>*Only common tests should be described solely by name; describe more complex techniques in the Methods section.*
☒	☐	A description of all covariates tested
☐	☒	A description of any assumptions or corrections, such as tests of normality and adjustment for multiple comparisons
☐	☒	A full description of the statistical parameters including central tendency (e.g. means) or other basic estimates (e.g. regression coefficient) AND variation (e.g. standard deviation) or associated estimates of uncertainty (e.g. confidence intervals)
☐	☒	For null hypothesis testing, the test statistic (e.g. *F*, *t*, *r*) with confidence intervals, effect sizes, degrees of freedom and *P* value noted<br>*Give P values as exact values whenever suitable.*
☒	☐	For Bayesian analysis, information on the choice of priors and Markov chain Monte Carlo settings
☒	☐	For hierarchical and complex designs, identification of the appropriate level for tests and full reporting of outcomes
☒	☐	Estimates of effect sizes (e.g. Cohen's *d*, Pearson's *r*), indicating how they were calculated

Our web collection on statistics for biologists contains articles on many of the points above.

Software and code

Policy information about availability of computer code

Data collection	For FACS analysis: FACSCelesta (BD) and Cytoflex (Beckman)<br>For qRT-PCR: Bio-Rad CFX Manager 3.1<br>For H&E staining and IHC: Leica Aperio VERSA 8<br>For multiplex immunohistochemical (mIHC) staining: Akoya Vectra3
Data analysis	GraphPad Prism 8 for graphs and statistical analysis<br>FlowJo-V10.6.2 for FACS plots<br>Bio-Rad CFX Manager 3.1 for qRT-PCR analysis<br>ImageJ 1.8.0 for Western Blot quantitation

For manuscripts utilizing custom algorithms or software that are central to the research but not yet described in published literature, software must be made available to editors and reviewers. We strongly encourage code deposition in a community repository (e.g. GitHub). See the Nature Portfolio guidelines for submitting code & software for further information.

Data

Policy information about availability of data

All manuscripts must include a data availability statement. This statement should provide the following information, where applicable:

- Accession codes, unique identifiers, or web links for publicly available datasets
- A description of any restrictions on data availability
- For clinical datasets or third party data, please ensure that the statement adheres to our policy

All data are available in the main text, supplementary information, or source data file. Further information and requests for reagents used in the study can be provided by contacting the corresponding authors.
All data are available in the main text, Supplementary Information, or source data file. The mass spectrometry proteomics data for identifying the potential PD-1-interacting proteins have been deposited to the ProteomeXchange Consortium (http://proteomecentral.proteomexchange.org) via the iProX partner repository with the dataset identifier PXD040671. Source data are provided with this paper.

Human research participants

Policy information about studies involving human research participants and Sex and Gender in Research.

Reporting on sex and gender	Three men and two women aged 52-74 years were enrolled from Zhongnan Hospital of Wuhan University. Given that a small sample size was included, no sex-and gender-based analyses have been performed.
Population characteristics	The samples are all from the same ethnicity. Asian.
Recruitment	All the patients were recruited at Zhongnan Hospital of Wuhan University. Informed consent was obtained by participants.
Ethics oversight	The use of pathological specimens and the review of all pertinent patient records were approved by the Research Ethics Committee of the Zhongnan Hospital of Wuhan University (Protocol # 2020106).

Note that full information on the approval of the study protocol must also be provided in the manuscript.

Field-specific reporting

Please select the one below that is the best fit for your research. If you are not sure, read the appropriate sections before making your selection.

[x] Life sciences [ ] Behavioural & social sciences [ ] Ecological, evolutionary & environmental sciences

For a reference copy of the document with all sections, see nature.com/documents/nr-reporting-summary-flat.pdf

Life sciences study design

All studies must disclose on these points even when the disclosure is negative.

Sample size	For in vitro experiments, three biologically independent experiments were conducted unless otherwise stated. Sample sizes were chosen based on previous publications, which were sufficient for statistical analysis (Zhang et al., Nature, 2018, 553(7686):91-95; Gao et al., Nat Cell Biol. 2020 Sep;22(9):1064-1075.).<br>For in vivo mice experiments, a sample size of n ≥ 4 mice per group was sufficient for statistical analysis and generating significant results based previous studies (Wang et al., Nature, 2017; 546(7658):426-430; Simoneschi et al., Nature, 2021; 592(7856):789-793.). In our mice experiments, sample sizes were chosen as large as possible and the exact number of mice used in each experiment were included in the figure legends.
Data exclusions	No data were excluded from the analysis.
Replication	All experiments results were reliably reproduced. The replication numbers were described in the corresponding figure legends.
Randomization	All samples were randomly allocated to each group as described in Methods.
Blinding	The investigators were not blinded to sample allocation during experiments because the information of the value of tumor size and different treatments among groups was essential and correctly to conduct the studies.

Reporting for specific materials, systems and methods

We require information from authors about some types of materials, experimental systems and methods used in many studies. Here, indicate whether each material, system or method listed is relevant to your study. If you are not sure if a list item applies to your research, read the appropriate section before selecting a response.

Materials & experimental systems

n/a	Involved in the study
	☒ Antibodies
	☒ Eukaryotic cell lines
☒	☐ Palaeontology and archaeology
	☒ Animals and other organisms
☒	☐ Clinical data
☒	☐ Dual use research of concern

Methods

n/a	Involved in the study
☒	☐ ChIP-seq
	☒ Flow cytometry
☒	☐ MRI-based neuroimaging

Antibodies

Antibodies used

The following is the information of antibodies used in immunoprecipitation (IP) and immunoblotting (IB). Anti-PD-1 (D4W2J) rabbit mAb (1:2000 dilution, Cat# 86163, RRID:AB_2728833), anti-PD-1 (Intracellular Domain) (D7D5W) rabbit mAb (1:2000 dilution, Cat# 84651, RRID:AB_2800041), anti-K48-linkage Specific Polyubiquitin rabbit pAb (1:1000 dilution, Cat# 4289, RRID:AB_10557239), anti-Phospho-p44/42 MAPK (Erk1/2) (Thr202/Tyr204) rabbit mAb (1:2000 dilution, Cat# 4370, RRID:AB_2315112), anti-Myc-Tag (71D10) Rabbit mAb (1:1000 dilution, Cat# 2278, RRID:AB_490778), and anti-USP9X (D4Y7W) rabbit mAb (1:2000 dilution, Cat# 14898, RRID:AB_2798640) were purchased from Cell Signaling Technology. Anti-USP5 rabbit pAb (1:5000 dilution, Cat# A4202, RRID:AB_2765553), anti-USP10 rabbit mAb (1:5000 dilution, Cat# A4454, RRID:AB_2863277), anti-USP24 rabbit pAb (1:5000 dilution, Cat# A20003, RRID:AB_2862911), anti-ERK1/2 rabbit mAb (1:2000 dilution, Cat# A4782, clone: ARC0212, RRID:AB_2863347), anti-rabbit Control IgG (Cat# AC005, RRID:AB_2771930), anti-mouse Control IgG (Cat# AC011, RRID:AB_2770414), and anti-Biotin rabbit mAb (1:3000 dilution, Cat# A20684) was purchased from ABclonal Technology. Anti-Vinculin (VIN-11-5) mouse mAb (1:100000 dilution, Cat# V4505, RRID:AB_477617), anti-Flag rabbit pAb (1:50000 dilution, Cat# F7425, RRID:AB_439687), anti-Flag M2 mouse mAb (1:50000 dilution, Cat# F3165, RRID:AB_259529), anti-HA rabbit pAb (1:50000 dilution, Cat# H6908, RRID:AB_260070), anti-HA Agarose (Cat# A2095, RRID:AB_257974), and anti-Flag M2 affinity gel (Cat# A2220, RRID:AB_10063035) were purchased from Sigma-Aldrich. Anti-PD-1/CD279 mouse mAb (1:2000 dilution, Cat# 66220-1-Ig, RRID:AB_2881611), anti-PD-1/CD279 rabbit pAb (1:2000 dilution, Cat# 18106-1-AP, RRID:AB_10732952), anti-USP5 mouse mAb (1:5000 dilution, Cat# 66213-1-Ig, RRID:AB_2881604), anti-USP15 rabbit pAb (1:5000 dilution, Cat# 14354-1-AP, RRID:AB_2257148), and anti-GAPDH mouse mAb (1:50000 dilution, Cat# 60004-1-Ig, RRID:AB_2107436) were purchased from Proteintech. Purified anti-HA.11 Epitope Tag Antibody (1:2000 dilution, Cat# 902301, RRID:AB_2565018) were purchased from Biolegend. Anti-GST tag mouse mAb (Cat# AT0027) was purchased from Engibody.
The rabbit polyclonal phosphorylation antibodies against pT234-PD-1 (1:5000) generated by Abclonal Technology were derived from rabbit with three clones. The antigen sequence for PD-1 used for immunization: 231-REK(p)TPEPP-238. (p)T stands for phosphorylated T234 residue in this synthetic peptide. The antibodies were purified using the antigen peptide column.
The following is the information of antibodies used in flow cytometry analysis. Anti-CD45-APC-Cy7 (1:100 dilution, Cat# 557659, RRID:AB_396774), anti-CD3-BV421 (1:50 dilution, Cat# 562600, RRID:AB_11153670), anti-CD8α-BV510 (1:50 dilution, Cat# 566096, RRID:AB_2739500), anti-CD4-BV605 (1:50 dilution, Cat# 563151, RRID:AB_2687549), anti-PD-1-PE (1:50 dilution, Cat# 551892, RRID:AB_394284), anti-IFN-γ-APC (1:20 dilution, Cat# 562303, RRID:AB_11153140), and anti-TNF-PE (1:20 dilution, Cat# 554419, RRID:AB_395380), anti-CD44- PerCp Cy5.5 (1:50 dilution, Cat# 560570, RRID:AB_1727486), anti-CD25-FITC (1:50 dilution, Cat# 564424, RRID:AB_2738803), anti-CD69-FITC (1:50 dilution, Cat# 553236, RRID:AB_394725), anti-IL10-PE (1:50 dilution, Cat# 554467, RRID:AB_395412), anti-TIM3-BV786 (1:50 dilution, Cat# 747621, RRID:AB_2744187), anti-CD62L-BV650 (1:50 dilution, Cat# 564108, RRID:AB_2738597), anti-Ki67-Alexa Fluor 647 (1:50 dilution, Cat# 561126, RRID:AB_10611874), anti-Foxp3-Alexa Fluor 647 (1:30 dilution, Cat# 560401, RRID:AB_1645201), and fixable viability stain 700 (1:300 dilution, Cat# 564997, RRID:AB_2869637) were purchased from BD Biosciences. Anti-PD-1-PE (1:50 dilution, Cat# 114117, RRID:AB_2566725), anti-PD-1-APC (1:50 dilution, Cat# 135210, RRID:AB_2159183), anti-CD28-PerCp Cy5.5 (1:50 dilution, Cat# 102114, RRID:AB_2073850), anti-CTLA-4-PE (1:50 dilution, Cat# 106305, RRID:AB_313254), and anti-TGF-β- PerCp Cy5.5 (1:50 dilution, Cat# 141409, RRID:AB_2561591) were purchased from Biolegend. Anti-GzmB (1:50 dilution, Cat# 61-8898-82, RRID:AB_2574670) was purchased from eBioscience.
The following antibodies were used for multiplexed immunohistochemistry (mIHC), IHC, or immunofluorescence. Anti-human CD3 (1:200 dilution, Cat# 85061, RRID:AB_2721019), anti-mouse CD3 (1:200 dilution, Cat# 99940, RRID:AB_2755035), anti-CK (1:400 dilution, Cat# 4545, RRID:AB_490860), anti-human PD-1 (1:400 dilution, Cat# 86163, RRID:AB_2728833), anti-mouse PD-1 (1:400 dilution, Cat# 84651, RRID:AB_2800041), anti-phospho-ERK1/2 (1:200 dilution, Cat# 4370, RRID:AB_2315112), anti-CD8α (1:400 dilution, Cat# 98941, RRID:AB_2756376) were purchased from Cell Signaling Technology. Anti-Granzyme B (1:800 dilution, Cat# ab4059, RRID:AB_304251), anti-CD4 (1:1500 dilution, Cat# ab183685, RRID:AB_2686917), and anti-Foxp3 (1:1500 dilution, Cat# ab215206, RRID:AB_2860568) were purchased from Abcam. Anti-USP5 rabbit pAb (1:1500 dilution, Cat# A4202, RRID:AB_2765553) and anti-ERK1/2 rabbit mAb (1:200 dilution, Cat# A4782, clone: ARC0212, RRID:AB_2863347) were purchased from ABclonal Technology. Anti-PD-1 (1:400 dilution, Cat# 66220-1-Ig, RRID:AB_2881611), anti-Calnexin (1:50 dilution, Cat# 10427-2-AP, RRID:AB_2069033), anti-Calnexin (1:50 dilution, Cat# 66903-1-Ig, RRID:AB_2882231), and anti-GM130 (1:50 dilution, Cat# 11308-1-AP, RRID:AB_2115327) were purchased from Proteintech.
For tumor mouse model analysis, antibodies we used in this study are below. Anti-mouse PD-1 (100 ug/mouse, clone: 29F.1A12) and anti-mouse CTLA-4 (100 ug/mouse, clone: 9H10) for treatment were provided by the Laboratory of Dr. Gordon J. Freeman.

Validation

All antibodies used in this study are commercially available and have been validated by manufacturer. Any validation statements are available on the manufacturer's website. The RRID# of each antidoies is also provided here.
The following antibodies were purchased from Cell Signaling Technology:
anti-PD-1 (D4W2J) rabbit mAb (1:2000 dilution, Cat# 86163, RRID:AB_2728833),
anti-PD-1 (Intracellular Domain) (D7D5W) rabbit mAb (1:2000 dilution, Cat# 84651, RRID:AB_2800041),
anti-K48-linkage Specific Polyubiquitin rabbit pAb (1:1000 dilution, Cat# 4289, RRID:AB_10557239),
anti-Phospho-p44/42 MAPK (Erk1/2) (Thr202/Tyr204) rabbit mAb (1:2000 dilution, Cat# 4370, RRID:AB_2315112),
anti-Myc-Tag (71D10) Rabbit mAb (1:1000 dilution, Cat# 2278, RRID:AB_490778), anti-USP9X (D4Y7W) rabbit mAb (1:2000 dilution, Cat# 14898, RRID:AB_2798640),

anti-human CD3 (1:200 dilution, Cat# 85061, RRID:AB_2721019),
anti-mouse CD3 (1:200 dilution, Cat# 99940, RRID:AB_2755035),
anti-CK (1:400 dilution, Cat# 4545, RRID:AB_490860),
anti-CD8α (1:400 dilution, Cat# 98941, RRID:AB_2756376)
The following antibodies were purchased from ABclonal:
anti-USP5 rabbit pAb (1:5000 dilution, Cat# A4202, RRID:AB_2765553),
anti-USP10 rabbit mAb (1:5000 dilution, Cat# A4454, RRID:AB_2863277),
anti-USP24 rabbit pAb (1:5000 dilution, Cat# A20003, RRID:AB_2862911),
anti-ERK1/2 rabbit mAb (1:2000 dilution, Cat# A4782, clone: ARC0212, RRID:AB_2863347),
anti-rabbit Control IgG (Cat# AC005, RRID:AB_2771930),
anti-mouse Control IgG (Cat# AC011, RRID:AB_2770414),
and anti-Biotin rabbit mAb (1:3000 dilution, Cat# A20684).
The following antibodies were purchased from Sigma-Aldrich:
anti-Vinculin (VIN-11-5) mouse mAb (1:100000 dilution, Cat# V4505, RRID:AB_477617),
anti-Flag rabbit pAb (1:50000 dilution, Cat# F7425, RRID:AB_439687),
anti-Flag M2 mouse mAb (1:50000 dilution, Cat# F3165, RRID:AB_259529),
anti-HA rabbit pAb (1:50000 dilution, Cat# H6908, RRID:AB_260070),
anti-HA Agarose (Cat# A2095, RRID:AB_257974),
anti-Flag M2 affinity gel (Cat# A2220, RRID:AB_10063035)
The following antibodies were purchased from Proteintech:
anti-PD-1/CD279 mouse mAb (1:2000 dilution, Cat# 66220-1-Ig, RRID:AB_2881611), anti-PD-1/CD279 rabbit pAb (1:2000 dilution, Cat# 18106-1-AP, RRID:AB_10732952),
anti-USP5 mouse mAb (1:5000 dilution, Cat# 66213-1-Ig, RRID:AB_2881604),
anti-USP15 rabbit pAb (1:5000 dilution, Cat# 14354-1-AP, RRID:AB_2257148),
and anti-GAPDH mouse mAb (1:50000 dilution, Cat# 60004-1-Ig, RRID:AB_2107436),
anti-PD-1 (1:400 dilution, Cat# 66220-1-Ig, RRID:AB_2881611),
anti-Calnexin (1:50 dilution, Cat# 10427-2-AP, RRID:AB_2069033),
anti-Calnexin (1:50 dilution, Cat# 66903-1-Ig, RRID:AB_2882231),
anti-GM130 (1:50 dilution, Cat# 11308-1-AP, RRID:AB_2115327).
The following antibodies were purchased from Biolegend:
Purified anti-HA.11 Epitope Tag Antibody (1:2000 dilution, Cat# 902301, RRID:AB_2565018).
anti-PD-1-PE (1:50 dilution, Cat# 114117, RRID:AB_2566725),
anti-PD-1-APC (1:50 dilution, Cat# 135210, RRID:AB_2159183),
anti-CD28-PerCp C1:50 dilution, γ5.5 (Cat# 102114, RRID:AB_2073850),
anti-CTLA-4-PE (1:50 dilution, Cat# 106305, RRID:AB_313254),
and anti-TGF-β- PerCp Cy5.5 (1:50 dilution, Cat# 141409, RRID:AB_2561591).
Anti-GST tag mouse mAb (Cat# AT0027) was purchased from Engibody.
The following antibodies were purchased from BD Biosciences:
anti-CD45-APC-Cy7 (1:100 dilution, Cat# 557659, RRID:AB_396774), anti-CD3-BV421 (Cat# 562600, RRID:AB_11153670),
anti-CD8α-BV510 (1:50 dilution, Cat# 566096, RRID:AB_2739500),
anti-CD4-BV605 (1:50 dilution, Cat# 563151, RRID:AB_2687549),
anti-PD-1-PE (1:50 dilution, Cat# 551892, RRID:AB_394284),
anti-IFN-γ-APC (1:20 dilution, Cat# 562303, RRID:AB_11153140),
and anti-TNF-PE (1:20 dilution, Cat# 554419, RRID:AB_395380),
anti-CD44- PerCp Cy5.5 (1:50 dilution, Cat# 560570, RRID:AB_1727486),
anti-CD25-FITC (1:50 dilution, Cat# 564424, RRID:AB_2738803),
anti-CD69-FITC (1:50 dilution, Cat# 553236, RRID:AB_394725),
anti-IL10-PE (1:50 dilution, Cat# 554467, RRID:AB_395412),
anti-TIM3-BV786 (1:50 dilution, Cat# 747621, RRID:AB_2744187),
anti-CD62L-BV650 (1:50 dilution, Cat# 564108, RRID:AB_2738597),
anti-Ki67-Alexa Fluor 647 (1:50 dilution, Cat# 561126, RRID:AB_10611874),
anti-Foxp3-Alexa Fluor 647 (1:30 dilution, Cat# 560401, RRID:AB_1645201),
and fixable viability stain 700 (1:300 dilution, Cat# 564997, RRID:AB_2869637)
The following antibodies were purchased fromAbcam:
anti-Granzyme B (1:800 dilution, Cat# ab4059, RRID:AB_304251),
anti-CD4 (1:1500 dilution, Cat# ab183685, RRID:AB_2686917),
and anti-Foxp3 (1:1500 dilution, Cat# ab215206, RRID:AB_2860568).
Anti-GzmB (1:50 dilution, Cat# 61-8898-82, RRID:AB_2574670) was purchased from eBioscience.
The rabbit polyclonal phosphorylation antibodies against pT234-PD-1 (1:5000) generated by Abclonal Technology were derived from rabbit with three clones. The antigen sequence for PD-1 used for immunization: 231-REK(p)TPEPP-238. (p)T stands for phosphorylated T234 residue in this synthetic peptide. The antibodies were purified using the antigen peptide column.

Eukaryotic cell lines

Policy information about cell lines and Sex and Gender in Research

Cell line source(s)	Following cell lines were used in this study: HEK293T (RRID:CVCL_0063), Jurkat (RRID:CVCL_0065), MOLT-4 (RRID:CVCL_0013), CT26 (RRID:CVCL_7254), MC38 (RRID:CVCL_B288), and E.G7-OVA (RRID:CVCL_3505). MC38 was gifted by Dr. Arlene H. Sharpe lab in Harvard Medical School. All other cell lines were originally purchased from American Type Culture

	Collection (ATCC).
Authentication	All cell lines were authenticated by the STR profiling analysis.
Mycoplasma contamination	All cell lines were tested negative for mycoplasma contamination.
Commonly misidentified lines (See ICLAC register)	No commonly misidentified cell lines were used.

Animals and other research organisms

Policy information about studies involving animals; ARRIVE guidelines recommended for reporting animal research, and Sex and Gender in Research

Laboratory animals	Usp5flox/flox mice were generated by Cyagen Biosciences on its Crisper/Cas9 Gene Targeting Platform. To generate this mouse, mouse fertilized eggs were injected with a mixture of Cas9, gRNA and targeting vector. Usp5flox/flox mice were then crossed with Cd4-Cre transgenic mice to obtain Usp5 cKO mice with Usp5 ablation in T cells.<br>For the lung tumor model in KrasG12D/+Tp53fl/fl (KP) mice, five weeks after tumor induction by intranasal instillation of Adenovirus-Cre, mice were randomized into indicated groups and treated with the USP5 inhibitor (5 mg/kg) and Trametinib (0.25 mg/kg).<br>For syngeneic mouse tumor model assays, 4-6 weeks old female BALB/c mice were purchased from Charles River Laboratory and used for MC38 or CT26 tumor cells implantation.<br>OT-1 C57BL/6 mice contain transgenic inserts for mouse Tcra-V2 and Tcrb-V5 genes. The transgenic T cell receptor was designed to recognize ovalbumin residues 257-264 in the context of H2Kb and used to study the role of peptides in positive selection and the response of CD8+ T cells to antigen. Like most TCR transgenics, these mice are somewhat immunodeficient.<br>All mice were maintained in pathogen-free facilities with a 12h light/dark cycle at 20°±3 and 40–50% humidityat the Medical Research Institute of Wuhan University.
Wild animals	No wild animals were used in this study.
Reporting on sex	For in vivo experimental therapy in syngeneic mouse tumor models in BALB/c mice, only female BALB/c mice were uesd. For the genetic mice models, including Usp5 cKO C57BL/6J mice and KrasG12D/+Tp53fl/fl (KP) mice, both male and female mice were applied.
Field-collected samples	No field-collected samples were used in the study.
Ethics oversight	The Institutional Animal Care and Use Committee of Wuhan University (Protocol #: MRI2021-LAC13, MRI2021-LAC56, and MRI2021-LAC020).

Note that full information on the approval of the study protocol must also be provided in the manuscript.

Flow Cytometry

Plots

Confirm that:

- [x] The axis labels state the marker and fluorochrome used (e.g. CD4-FITC).
- [x] The axis scales are clearly visible. Include numbers along axes only for bottom left plot of group (a 'group' is an analysis of identical markers).
- [x] All plots are contour plots with outliers or pseudocolor plots.
- [x] A numerical value for number of cells or percentage (with statistics) is provided.

Methodology

Sample preparation	The single-cell suspensions were generated in PBS buffer with 1% FBS and 0.1 mM EDTA. Cells were incubated with APC-conjugated anti-human PD-1 antibody (Clone EH12.2H7, Biolegend) for 30 min at 4°C in the dark, then the cells were washed twice with PBS buffer containing 1% FBS and 0.1 mM EDTA. For apoptosis analysis, cells were washed once with PBS and stained with Annexin V and propidium iodide in 1× binding buffer for 10 min at the room temperature in the dark place. Flow cytometry data were acquired on a FACS flow cytometer (Beckman, Cytoflex) and analyzed with Flowjo 10.6.2 software (TreeStar).
Instrument	Beckman Cytoflex and BD FACSCelesta
Software	Flowjo 10.6.2 software (TreeStar).
Cell population abundance	For Analysis of membrane PD-1, cells that were not stained with APC or PE conjugated PD-1 were used as negative control and the proportion of APC or PE cells was 0%. The proportion of stained cells were 30% - 90% in experimental or control groups. Each group consisted of at least 10^6 cells.<br>For tumor infiltrated immune cells analysis with flow cytometry: cells that were not stained with conjugated antibodies as negative control and the proportion of this cells were 0%. The proportion of stained cells varies with the target cell populations. Each group of sorted cells was at least 10^5 cells.

Gating strategy	Forward versus side scatter(FSC vs SSC) gating was used to identify cells and exclude cell debris and dead cells. A forward scatter (FCS-H) vs. forward scatter area (FCS-A) density plot was used to exclude doublets. In some experiments, dead cells were further gated out on SSC-A and live/dead staining. Cytokine expression were detected in the populations of CD45+CD3 +ells. Or the target cell populations for further analysis were gated by cell surface marker.Gate boundaries were set either based on control samples (fluorescence minus-one or flourescence-minus-two controls), or followed density distributions based on best practices.

☒ Tick this box to confirm that a figure exemplifying the gating strategy is provided in the Supplementary Information.
