# Supplementary figures and images for: Author Correction: ERK and USP5 govern PD-1 homeostasis via deubiquitination to modulate tumor immunotherapy
Source: Nat Commun. 2025 Jun 20;16:5357. doi: 10.1038/s41467-025-61049-w (PMC12181289; doi:10.1038/s41467-025-61049-w)

Original Fig. 6d

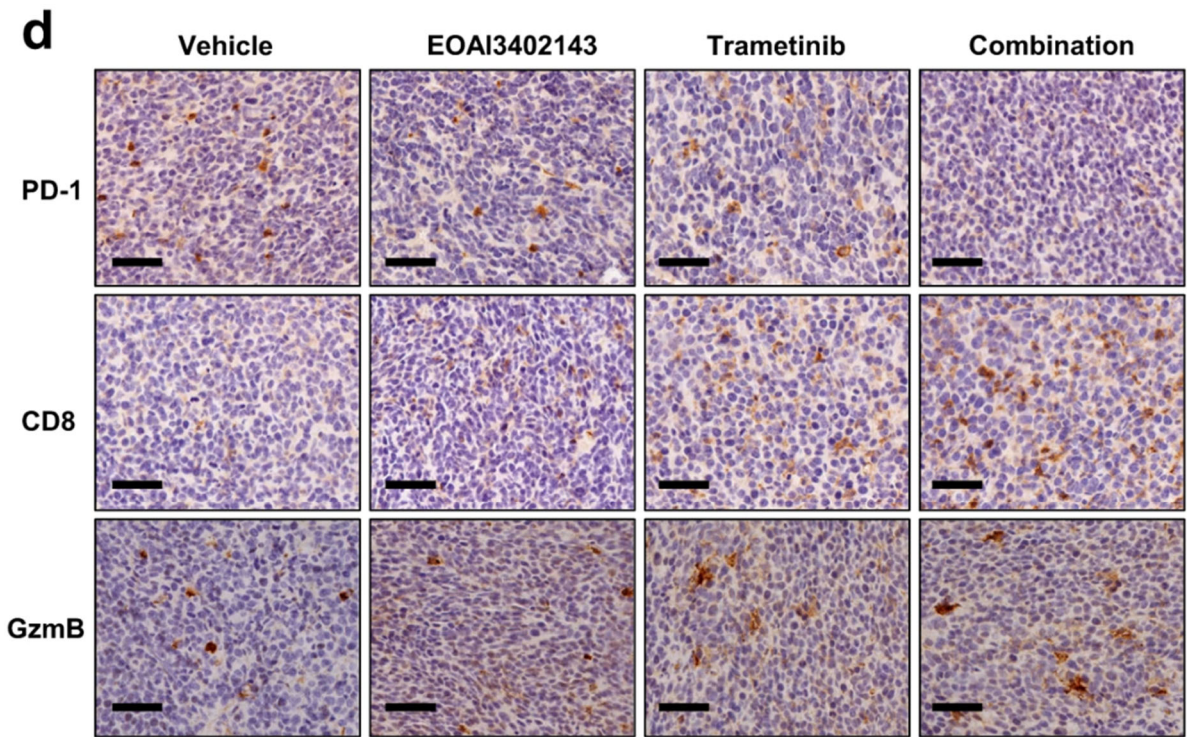

Original Supplementary Fig. 6d

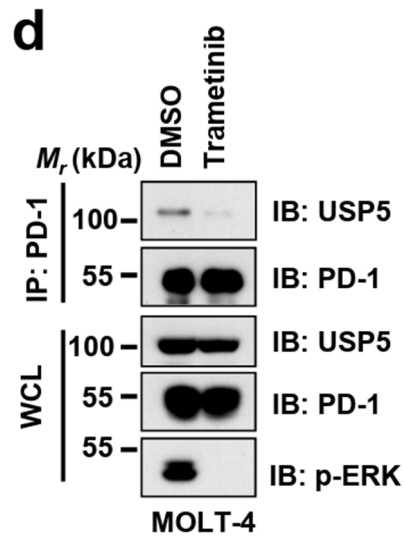

Original Supplementary Fig. 10b

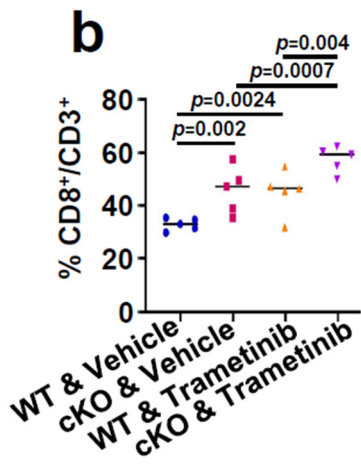

Supplement: Supplementary file 1 — Original, incorrect figures [file 41467_2025_61049_MOESM1_ESM.pdf]
